# Dried Blood Spot for CXCL-10 and Tacrolimus: Integrated Non-Invasive Monitoring to Guide Personalized Treatment in Adult Kidney Transplant Recipients

**DOI:** 10.3390/ph19020292

**Published:** 2026-02-10

**Authors:** Olga Millán, Jordi Rovira, Virginia Fortuna, Pedro Ventura-Aguiar, Fritz Diekmann, Mercè Brunet

**Affiliations:** 1 Biomedical Research Center in Hepatic and Digestive Diseases (CIBERehd), Instituto de Salud Carlos III, 28029 Madrid, Spain; omillan@clinic.cat; 2 Pharmacology and Toxicology, Biochemistry and Molecular Genetics, Biomedical Diagnostic Center (CDB), Hospital Clinic of Barcelona, Institut d’Investigacions Biomèdiques August Pi i Sunyer (FRCB-IDIBAPS), University of Barcelona, 08036 Barcelona, Spain; vfortuna@clinic.cat; 3 Nephrology and Transplantation (LENIT), Fundació Recerca Clínic Barcelona-Institut d’Investigacions Biomèdiques August Pi i Sunyer (FRCB-IDIBAPS), 08036 Barcelona, Spain; jrovira1@recerca.clinic.cat (J.R.); pventura@clinic.cat (P.V.-A.); 4 RICORS (RD21-0005-0003), Instituto de Salud Carlos III, 28029 Madrid, Spain; 5 Department of Nephrology and Kidney Transplantation, ICNU, Hospital Clínic de Barcelona, 08036 Barcelona, Spain

**Keywords:** dried blood spot, CXCL-10, tacrolimus, kidney transplantation, therapeutic drug monitoring, CMV infection, rejection, pharmacodynamic biomarkers, personalized immunosuppression

## Abstract

**Background/objectives:** Kidney transplant recipients require lifelong immunosuppression and monitoring to prevent rejection, infection, and graft dysfunction. Current surveillance relies on tacrolimus therapeutic drug monitoring and, when needed, invasive biopsies. Dried blood spot (DBS) sampling provides a minimally invasive, patient-friendly option for remote follow-up. This study aims to develop and evaluate a DBS-based method for CXCL-10 quantification that, in combination with tacrolimus exposure monitoring, could help identify kidney recipients at risk of rejection and cytomegalovirus (CMV) infection and guide immunosuppression adjustment. **Methods:** The study included 81 selected kidney recipients for CXCL-10-DBS analysis by ELISA (12 T-cell mediated rejection; 10 antibody-mediated rejection; 6 CMV infection and 53 clinical event-free) and 10 healthy volunteers. A Tacrolimus-DBS LC-MS/MS method was developed and validated, and it was compared with the reference method on venous whole blood (WB) LC-MS/MS in a validation cohort (n = 160) and a clinical cohort (n = 36) using linear regression, Passing–Bablok and Bland–Altman analyses. **Results:** CXCL-10-DBS concentrations were significantly higher in rejectors (*p* < 0.001), with intermediate increases in CMV infection in comparison with event-free patients and healthy volunteers. ROC analysis demonstrated excellent diagnostic accuracy for rejection (AUC: 0.952; cutoff: 216.2 pg/mL; sensitivity: 100%; specificity: 79%; PPV: 88%; NPV: 100%). In contrast, tacrolimus trough concentrations did not differ significantly among the three clinical groups but showed strong correlation and agreement between DBS and venous WB with no systematic or proportional bias. **Conclusions**: This pilot study demonstrates the feasibility and diagnostic potential of DBS-based CXCL-10 measurement in adult kidney recipients. Integration of DBS-tacrolimus monitoring supports a minimally invasive pharmacokinetic–pharmacodynamic approach for personalized immunosuppression management.

## 1. Introduction

Kidney transplantation is widely recognized as the optimal treatment for patients with end-stage renal disease, as it provides substantial advantages in terms of patient survival and quality of life when compared with dialysis [1]. Nevertheless, kidney transplant recipients typically receive multiple concomitant medications and require lifelong immunosuppressive therapy to maintain allograft function. Although indispensable, long-term immunosuppression is associated with an increased burden of comorbidities relative to the general population, including metabolic, cardiovascular, and infectious complications, as well as progressive graft dysfunction [2,3]. Together, these factors critically influence both long-term graft outcomes and overall patient survival.

The clinical complexity inherent to kidney transplantation highlights the need for monitoring strategies that are both personalized and easy to implement in routine care and that reflect not only drug exposure (pharmacokinetics) but also the biological effects of immunosuppression in the immune system response (pharmacodynamics). Within this framework, dried blood spot (DBS) sampling has gained increasing interest as a minimally invasive and cost-efficient approach that enables decentralized or home-based monitoring [4]. By requiring only a small volume of capillary blood, DBS facilitates the concurrent evaluation of pharmacokinetic markers, such as tacrolimus concentrations [5,6,7], together with pharmacodynamic and clinical biomarkers, including cytokines, chemokines, and creatinine [8,9,10]. This integrated assessment may provide a more comprehensive overview of the patient’s immunological activity and therapeutic status.

The Immunosuppressive Drugs Scientific Committee of the International Association of Therapeutic Drug Monitoring and Clinical Toxicology recently published a consensus report on therapeutic drug monitoring (TDM) for personalized tacrolimus therapy [11]. This report underscores the importance of a holistic approach to pharmacokinetic monitoring, one that integrates pharmacodynamic and pharmacogenetic biomarkers, to achieve truly individualized treatment. One of the central issues discussed was whether C_0_ remains the most appropriate parameter for routine tacrolimus TDM. Given the prevailing opinion that C_0_ is easy to measure in clinical practice, correlates reasonably well with the area under the curve (AUC), and has been associated with excellent short-term outcomes in renal transplant recipients, it is challenging to persuade clinicians that more labour-intensive strategies, such as AUC-guided monitoring, may be required to optimize long-term patient and graft outcomes [12,13]. The specific patient groups in which AUC monitoring should be implemented remain a matter of debate. It has been proposed that patients in the early post-transplant period (7–10 days after transplantation), before immunosuppression minimization, and particularly those with a low C_0_/dose ratio—who may exhibit high peak concentrations and elevated tacrolimus AUC—are the most likely to benefit. The primary anticipated advantage is improved renal function [11,14].

In line with evolving TDM requirements, several studies have validated DBS-based methods for tacrolimus quantification (DBS-TAC) in capillary blood. These studies demonstrate strong concordance with conventional venous whole blood (WB) measurements while offering notable logistical advantages related to sample collection, transportation, and storage. Such properties make DBS particularly appealing for longitudinal TDM in transplant recipients [15,16]. Clinical validation studies further indicate that DBS derived results are interchangeable with those obtained from the standard matrix used for tacrolimus TDM, namely WB [17].

Concurrently, extensive research in the field of transplant immunology has established the relevance of chemokines measured in plasma or urine as non-invasive indicators of kidney allograft inflammation [18,19,20,21]. Among these, CXCL-10 has emerged as one of the most extensively studied biomarkers. The incorporation of CXCL-10 into diagnostic algorithms and molecular scores has shown promise for both the prediction and diagnosis of T-cell–mediated rejection (TCMR) and antibody-mediated rejection (ABMR), supporting individualized immunosuppression adjustment and improved prevention of acute and chronic rejection [22,23,24,25].

CXCL-10 is an interferon-γ-inducible chemokine involved in the recruitment of activated T lymphocytes and other immune effector cells to sites of allograft inflammation, thereby sustaining and amplifying alloimmune responses. Elevated CXCL-10 levels have been consistently associated with active alloimmune injury, reflecting subclinical inflammation before histological or functional graft deterioration. Its clinical utility as a non-invasive biomarker of rejection and graft outcome has been validated in multiple studies using plasma and urine matrices [26]. Rabant et al. [27] demonstrated that urinary CXCL-10, corrected for creatinine, predicts rejection development with a high negative predictive value (NPV) and Mačionienė et al. [18] recently showed that CXCL-10/creatinine was able to distinguish between patients with transplant rejection and those without rejection. Our group [20] further showed that plasma CXCL-10 improves pre-transplant risk stratification and post-transplant rejection prediction with sensitivity and specificity above 80%. Mülbacher et al. [28] reported higher CXCL-10 levels in donor-specific antibody-positive recipients with ABMR. More recently, Hirt-Minkowski et al. [29] conducted the first randomized clinical trial (NCT03140514), confirming the biomarker’s diagnostic utility, though without significant improvement in one-year clinical outcomes. A second ongoing multicenter RCT (NCT03206801) aims to further clarify the clinical impact of CXCL-10 guided monitoring.

To date, relatively few studies have explored the feasibility and clinical utility of measuring CXCL-10 using dried blood or plasma spot-based approaches. Most available evidence originates from non-transplant clinical contexts, particularly infectious and inflammatory diseases, such as tuberculosis [30,31] and autoimmune disorders [32], where CXCL-10 quantified in dried plasma spots (DPS) or DBS has been investigated as a marker of immune activation and treatment response. Villar-Hernández et al. [33] and Hoel et al. [34] demonstrated that CXCL-10 concentrations measured in DPS strongly correlated with conventional plasma levels (r = 0.897), supporting the analytical reliability of this approach.

Despite these advances, the application of CXCL-10 measurement using DBS has not been evaluated in the field of solid organ transplantation, and its application in the kidney transplant setting remains unexplored. To our knowledge, no studies have assessed CXCL-10 in capillary WB DBS samples for the purpose of monitoring alloimmune activity or infectious complications in this population. The use of DBS technology offers an attractive alternative, enabling the simultaneous assessment of pharmacokinetic and pharmacodynamic biomarkers from a single, minimally invasive sample, while also actively involving patients in their own treatment monitoring through self-sampling.

The present pilot study aims to develop and evaluate a method for the quantification of CXCL-10 in DBS using ELISA methodology that, in combination with tacrolimus measurement by DBS-LC-MS/MS, would help in immunosuppression adjustment and would identify adult kidney transplant recipients at risk for allograft rejection (TCMR or ABMR) and/or cytomegalovirus (CMV) infection.

## 2. Results

### 2.1. Study Patients for CXCL-10 Analysis

In total, 81 adult kidney transplant recipients at the Renal Transplant Unit of the Hospital Clinic from February 2022 to July 2023 were included in this study. Clinical, demographic, and laboratory data are summarized in Table 1. Specifically, 53 patients free of clinical events, 12 patients with TCMR, 10 patients with ABMR, (all of them confirmed by biopsy), and 6 patients with CMV infection (CMV infection was defined as CMV DNA > 1000 copies/mL) were selected. An additional 10 healthy volunteers (NHC) were included.

Samples were collected prior to immunosuppressant administration (pre-dose). In cases of suspected rejection, samples were obtained before any modification of immunosuppressive therapy was implemented.

### 2.2. CXCL-10 Detection in Dried Blood Spots

CXCL-10 concentrations in DBS samples were measured using ELISA (see Section 4). The analytical performance of the CXCL-10 assay developed for DBS samples demonstrated satisfactory sensitivity and precision. The lower limit of quantification (LLOQ) was 1.67 pg/mL, ensuring reliable detection of CXCL-10 concentrations within the clinically relevant range. The intra-assay precision was 4.8%, and the inter-assay precision was 10.5%, both well within the generally accepted coefficient of variation (CV) limits for bioanalytical methods [≤15% for precision and ≤20% at the LLOQ, according to European Medicines Agency (EMA) guideline].

**Figure 1:**
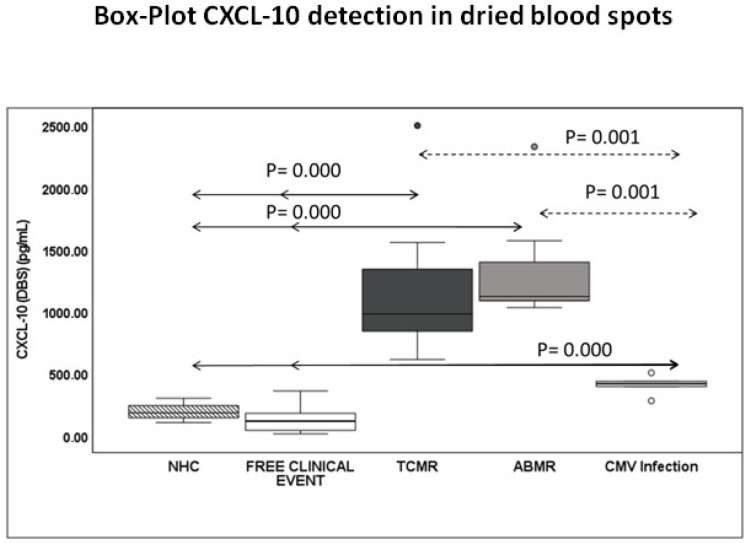
Box-Plot CXCL-10 detection in DBS. Box-plots showing differences in DBS CXCL-10 production among healthy volunteers (NHC, n = 10) (striped boxes); patients without clinical events (n = 53) (white boxes); patients with TCMR (n = 12) (black boxes), patients with ABMR (n = 10) (grey boxes) and those with CMV infection (n = 6) (light grey boxes). Boxes represent median and interquartile range. A value of *p* < 0.05 was considered to indicate statistical significance.

CXCL-10-DBS capillary blood concentrations were evaluated across different clinical conditions. Clinically stable kidney transplant recipients free of any clinical event showed a median CXCL-10 level of 122.9 pg/mL [IQR 41.52, 182], which was comparable to that observed in healthy controls (161.2 pg/mL [IQR 102.61, 199.58]; *p* > 0.05). In contrast, patients with biopsy-proven rejection displayed a marked and statistically significant increase in DBS-CXCL-10 levels, both in TCMR (1143.2 pg/mL [IQR 839.09, 1431.32]; *p* < 0.001) and ABMR (1305.7 pg/mL [IQR 1077.18, 1439.85]; *p* < 0.001) compared with stable recipients. Similarly, patients with active CMV infection showed a significant rise in DBS CXCL-10 concentrations (407.8 pg/mL [IQR 364.77, 453.36]; *p* < 0.001 vs. stable patients), although the magnitude of increase was lower than that observed in rejection cases. Differences between rejectors patients (TCMR or ABMR) and patients with CMV infection were also significant (*p* = 0.001) (Figure 1). Compared with event-free patients, CXCL-10 production in DBS increased approximately 9.3-fold in TCMR, 10.6-fold in ABMR, and 3.3-fold in patients with CMV infection.

**Figure 2:**
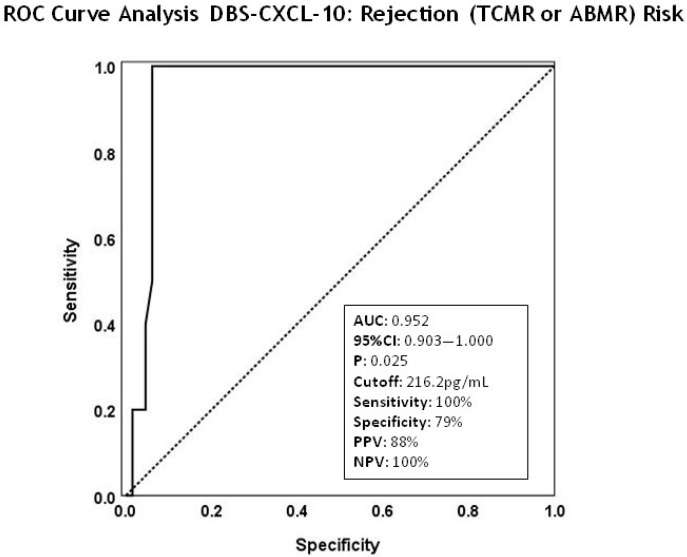
ROC curve analysis DBS-CXCL-10. ROC curve analysis for discrimination between rejectors (TCMR or ABMR) and non-rejectors for DBS-CXCL-10 concentration was evaluated. Diagnostic performance for identifying rejection [TCMR (n = 12) + ABMR (n = 10); total (n = 22)]: area under the ROC curve (AUCROC); 95% confidence interval (95% CI); cut-off value (pg/mL); Sensitivity (%); Specificity (%); PPV (%) and NPV (%).

A cutoff value for diagnosis rejection (TCMR or ABMR) was determined using ROC curve analysis of DBS CXCL-10 concentrations (Figure 2). The assay demonstrated an excellent diagnostic ability to discriminate between rejector patients and those free of clinical events (AUC = 0.952; 95% CI: 0.903–1.000). The optimal cutoff value for identifying rejection risk was 216.2 pg/mL, yielding a sensitivity of 100%, specificity of 79%, positive predictive value (PPV) of 88%, and negative predictive value (NPV) of 100%.

### 2.3. Tacrolimus Concentrations Measured in Whole Blood and DBS Across Clinical Groups

Tacrolimus concentrations in WB were determined using the LC-MS/MS method routinely implemented in our laboratory for clinical TDM. This method had been previously developed and validated by our group and therefore was used here exclusively as the reference method for comparison with DBS measurements [35].

**Figure 3:**
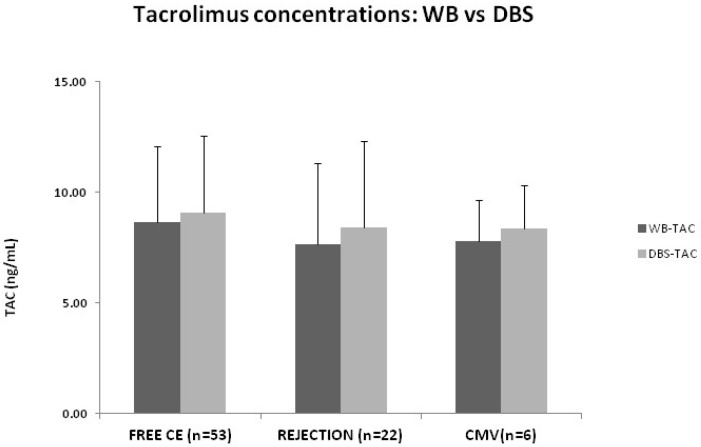
Tacrolimus concentrations measured in WB and DBS across clinical groups. Tacrolimus concentrations in whole blood (WB-TAC) and dried blood spots (DBS-TAC) in kidney transplant recipients classified as stable (free of clinical events, n = 53), patients with biopsy-proven rejection (TCMR or ABMR, n = 22), and patients with active CMV infection (n = 6). Bars represent mean ± standard deviation (SD).

Tacrolimus trough (C_0_) concentrations measured by the new validated DBS method (see Section 2.4) and in WB were comparable across all clinical groups: free clinical events, patients with rejection (TCMR or ABMR), and those with active CMV infection, indicating similar drug exposure (Figure 3). No significant differences (*p* > 0.05) were observed in tacrolimus levels between WB and DBS within each group, confirming the analytical equivalence of both matrices for TDM.

### 2.4. Tacrolimus DBS Method Validation

Paired DBS and WB samples were obtained in parallel for all participants. The validation of the method for DBS was performed according to the European Medicines EMA Guideline on bioanalytical method validation [36].

#### 2.4.1. Validation of the DBS Method

##### Calibration Model

Calibration analytical linearity was assessed once a day for 4 days using tacrolimus calibrators and blank [free of tacrolimus and internal standard (IS)-tacrolimus] and zero (free of tacrolimus) samples, all spiked to a large volume of blood from a single source, collected daily (>3 h drying). The method was linear over the calibration range from 1 to 45 ng/mL. All back-calculated concentrations of the calibrators fell within the acceptance criteria, and the regression determination coefficients (r^2^) were >0.99.

##### Carry Over

Carry over was assessed over 3 days by injecting singular extracted blanks after the highest calibration standard (ULOQ). The mean ratio between the peak areas measured in the blank extracts and the peak areas in the LLOQ was 8.3% (<20%) for tacrolimus and 2.7% (<5%) for ^13^Cd2-Tacrolimus.

##### Specificity

The analytical method demonstrated high specificity for the determination of tacrolimus DBS samples. The response obtained for potential comedications/analytes was less than 20% of the limit of quantification (LQ), while the response for the IS was less than 5%. Therefore, we could affirm that the analytical method is specific for the determination of tacrolimus.

##### Sensitivity (LQ)

The LLOQ (1.0 ng/mL) was accepted, as both accuracy and precision at this concentration were within ±20%, fulfilling regulatory acceptance criteria.

##### Intraday and Interday Accuracy and Precision

Intraday accuracy and precision were evaluated using DBS samples collected from WB from six different sources, spiked with tacrolimus at the LLOQ, 3 ng/mL (Quality Control Low, QCL), 7.5 ng/mL (Quality Control medium, QCM), and 15 ng/mL (Quality Control High, QCH), then extracted and analyzed in singular (one day).

The interday accuracy and precision were determined using DBS samples collected from a single source of WB spiked at the same time and concentrations levels (LLOQ, QCL, QCM, QCH). Duplicates at each concentration level were extracted and analyzed each day for three working days.

The intraday and interday precision, expressed as the coefficient of variation (CV%), were less than 9% (acceptable if the mean concentration fell within 20% of the nominal values for the LLOQ and 15% for all QC nominal concentrations), indicating excellent repeatability and intermediate precision of the method. The intraday and interday accuracy values were within ±12%, confirming that the method provides reliable and reproducible quantification of tacrolimus in DBS samples.

Detailed accuracy and precision results are provided in Supplementary Table S1.

##### Matrix Effect, Recovery and Hematocrit Effect

The matrix effect and recovery were evaluated simultaneously at the QCL and the QCH concentrations in DBS spotted with six different lots of WB, one of which was diluted or concentrated to obtain three levels of hematocrit (low 26%, medium 35%, and high 55%). Recovery was assessed by comparing DBS extracts of spiked WB, and the matrix effect was evaluated against pure solutions (n = 5). All were analyzed in quadruplicate. The matrix effect and the recovery showed acceptable results considering that the obtained relative standard deviation at each concentration was <15%, between matrices and between hematocrit values. With more detail, the relative standard deviation obtained for matrix effect ranged from 7.9 ng/mL to 11.3 ng/mL, whereas recovery ranged from 6.3 ng/mL to 10.4 ng/mL.

The hematocrit effect was evaluated by analyzing quadruplicates of DBS spotted with the spiked WB samples prepared at three levels of hematocrit used for the evaluation of the matrix effect and recovery. The back-calculated concentrations of tacrolimus in DBS spotted with low-to-high-hematocrit blood derived less than ±15% of medium-hematocrit blood.

Overall, these results demonstrate that the DBS-based LC–MS/MS method for tacrolimus quantification meets international validation standards, ensuring its suitability for clinical and pharmacokinetic applications.

### 2.5. Clinical Validation: Correlation Tacrolimus DBS vs. WB

To evaluate the correlation between tacrolimus DBS and WB (reference method), a cohort of 160 samples from adult stable renal transplant recipients was analyzed. DBS samples were prepared as described in Section 4.3.1. The patients were stable kidney transplant recipients (>12 months post-transplant) from deceased or living donors who received tacrolimus as an immunosuppressive treatment to prevent rejection.

**Figure 4:**
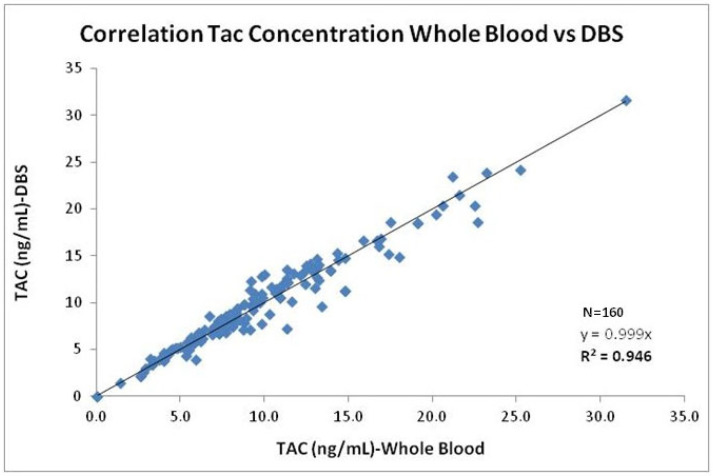
Correlation of tacrolimus concentration in whole blood vs. DBS. Evaluation of the correlation between DBS and WB venous blood concentrations for tacrolimus (n = 160 stable kidney transplant recipients).

**Figure 5:**
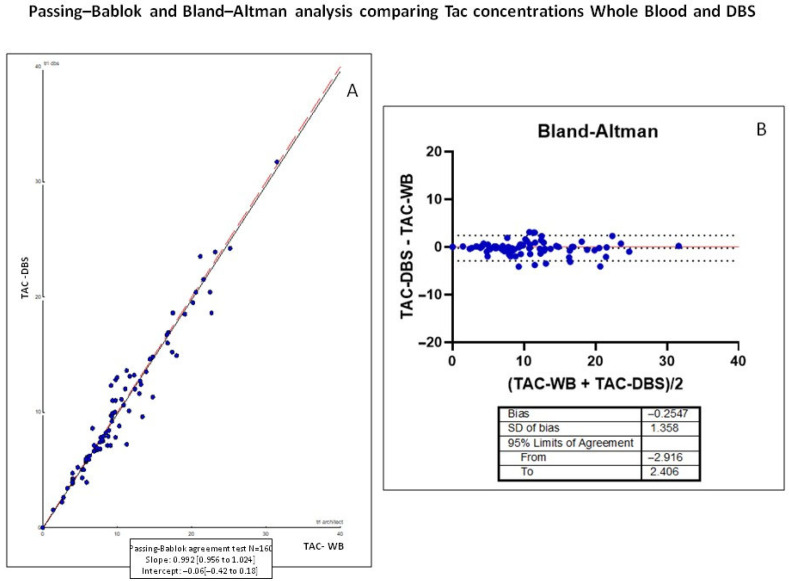
Passing–Bablok regression and Bland–Altman analysis assessing agreement between tacrolimus concentrations measured in DBS and WB. To evaluate agreement between matrices and assess potential systematic or proportional bias a Passing–Bablok (**A**) and a Bland–Altman (**B**) analysis was performed in the validation cohort. The solid line represents regression fit, and the dashed line corresponds to the line of identity (y = x).

As shown in Figure 4, tacrolimus concentrations measured in DBS correlated strongly with those obtained from WB, with a strong determination coefficient (r^2^ = 0.940). The linear regression analysis confirmed a good agreement between the two matrices, with most paired values being within the 95% limits of agreement and only a slight proportional bias observed at higher concentrations. These results support the analytical reliability and linearity of the DBS-LC-MS/MS method compared with the conventional WB assay. To complement the linear regression analysis shown in Figure 4, and to further assess the agreement between both matrices in the validation cohort, a Passing–Bablok and Bland–Altman analysis was performed (Figure 5). The Passing–Bablok regression, which is robust against outliers and does not assume normal distribution, provided an additional evaluation of method comparability. The resulting slope (0.992; 95% CI: 0.956–1.024) and intercept (−0.06; 95% CI: −0.42 to 0.18) indicated the absence of proportional or systematic bias between WB and DBS measurements. Bland–Altman analysis provided complementary information on agreement between methods, revealing a small mean bias of −0.2547 ng/mL. The standard deviation of the bias was 1.36 ng/mL, and the 95% limits of agreement ranged from −2.196 to 2.406 ng/mL. Importantly, no trend toward increasing bias across the concentration range was observed. These results demonstrate a good agreement between the two sampling methods, with no clinically relevant systematic bias observed. These findings support the analytical interchangeability of both matrices and confirm that DBS is a valid and reliable alternative for TDM.

#### Clinical Study Cohort (n = 36)

A new cohort was included, comprising 36 clinically stable kidney transplant recipients under maintenance immunosuppressive therapy (>12 months post-transplant) from deceased or living donors who received tacrolimus as an immunosuppressive treatment to prevent rejection.

Two DBS sampling modalities were evaluated in this group: nurse-collected samples (n = 20) and patient self-collected samples (n = 16) (detailed procedures are described in Section 4).

When analyzed individually, both sampling approaches showed excellent correlation and agreement between DBS and WB tacrolimus concentrations analyses confirming minimal bias and consistent distribution across the concentration range (nurse-collected r^2^ = 0.9404; self-patients collected r^2^ = 0.9315).

**Figure 6:**
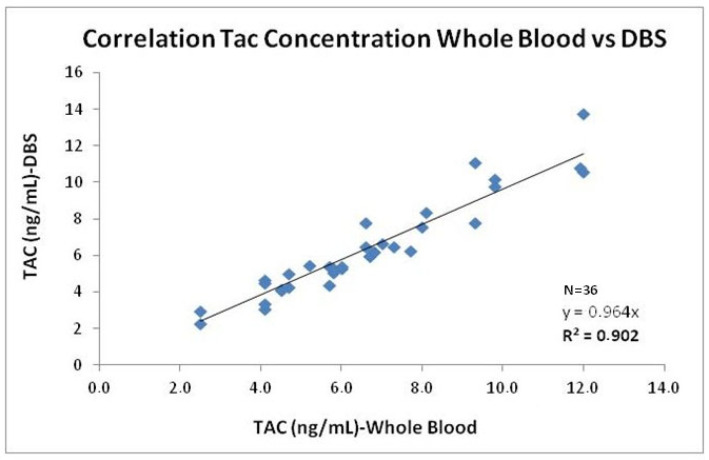
Correlation tacrolimus Concentration Whole Blood vs. Capillary Blood DBS. Evaluation of the correlation between DBS and WB venous blood concentrations for tacrolimus [n = 36: nurse-collected samples (n = 20) and self-patients collected samples (n = 16)].

Since both modalities showed strong and comparable correlations between DBS and WB tacrolimus concentrations, the two datasets were combined to increase the sample size and strengthen the statistical power of the analysis. The combined dataset (n = 36) maintained a high correlation (r^2^ = 0.902) and good agreement, as shown in Figure 6, confirming that DBS results are consistent regardless of the sampling modality.

## 3. Discussion

This study demonstrates the feasibility of quantifying CXCL-10 in capillary WB using DBS sampling in kidney transplant recipients. While CXCL-10 measurement in DBS has been previously reported in non-transplant settings [30,31,32], the present study is, to our knowledge, the first to extend this approach to the field of kidney transplantation, and more specifically to kidney transplant recipients. Although associations between tacrolimus exposure and CXCL-10 levels have previously been described using conventional matrices such as plasma or urine [21], this study integrates CXCL-10 assessment with tacrolimus determination from a single DBS sample. By combining CXCL-10 determination with tacrolimus measurement in DBS, this study proposes a minimally invasive and patient-friendly strategy for the simultaneous monitoring of pharmacokinetic and pharmacodynamic biomarkers, providing a broader view of a patient’s immunological and therapeutic status.

In the present study, CXCL-10 capillary blood concentrations measured in DBS showed a distinct distribution among adult transplant recipients, identifying a progressive increase from those with CMV infection to those with active rejection (TCMR or ABMR). Patients with rejection showed markedly elevated CXCL-10 levels compared to stable patients and healthy controls. In our cohort, DBS-CXCL-10 showed an excellent diagnostic performance for identifying biopsy-proven rejection. ROC curve analysis yielded an AUC of 0.952 (95% CI: 0.903–1.000), indicating outstanding discriminative accuracy. The optimal cutoff value was 216.2 pg/mL, which provided 100% sensitivity, 79% specificity, 88% PPV, and 100% NPV for distinguishing rejector patients from clinically stable recipients. Comparison with previously published cutoffs reinforces the biological consistency of these findings. In our earlier work [20], CXCL-10 thresholds of 177.7 pg/mL in plasma and 87.85 pg/mL in urine were associated with a high predictive capacity for subsequent rejection events. Although those studies assessed prediction rather than diagnosis, the relative increase across matrices (urine < plasma < DBS) is compatible with the known distribution of CXCL-10 and the higher cellular content of WB. Other groups have also shown similar CXCL-10 thresholds for detecting alloimmune injury [22,27,29]. Reported cutoffs vary depending on the biological matrix and assay format, typically ranging from low tens of pg/mL in urine to several hundred pg/mL in plasma or serum, consistent with our DBS threshold. Collectively, these data confirm that CXCL-10 elevations associated with rejection remain detectable and clinically meaningful irrespective of the sampling matrix used. Importantly, the magnitude of CXCL-10 elevation differed markedly between clinical conditions. Compared with event-free recipients, DBS-CXCL-10 concentrations increased approximately 9.3-fold in TCMR and 10.6-fold in ABMR, whereas patients with CMV infection exhibited a more moderate 3.3-fold increase. This quantitative gradient further supports the potential of CXCL-10 to aid in the differentiation of alloimmune-mediated rejection from virus-associated inflammatory responses, provided it is interpreted within the appropriate clinical context.

Patients with CMV infection exhibited intermediate increases, consistent with the inflammatory activation described in viral infections. Viral infections, particularly CMV and BK virus (BKV), are recognized as clinical confounding factors in the interpretation of chemokine-based biomarkers, especially as predictive or diagnostic biomarkers of rejection risk. Elevated plasma CXCL-10 levels can also occur in the setting of active viral infections, particularly CMV and BKV (although BKV infection was not evaluated in the present cohort) [37]. Several studies [20,38] have reported higher serum CXCL-10 concentrations in patients with normal histology, but concomitant CMV infection, compared with those without infection. Similarly, urinary CXCL-10 levels have been shown to increase in patients with BKV infection [22,39]. In our study, the magnitude of CXCL-10 elevation measured in DBS from patients with CMV infection was significantly lower than that observed in rejection cases, a finding consistent with previous reports using plasma samples. This observation further confirms the biological validity of CXCL-10 as a biomarker of alloimmune activation. Consequently, CXCL-10 quantification could serve as a complementary tool in the management of kidney transplant recipients; specifically, integrating CXCL-10 measurements with routine viral load testing may improve diagnostic accuracy and facilitate the clinical distinction between these two clinical entities. Importantly, the ROC-derived cutoff value identified in this study (216.2 pg/mL) was optimized to discriminate patients with rejection from those free of clinical events, prioritizing sensitivity and NPV. This cutoff is not intended to differentiate rejection from CMV infection. Given the limited number of CMV cases in our cohort (n = 6), performing a dedicated ROC analysis comparing rejection vs. CMV infection would not be statistically robust and could lead to unreliable estimates. Therefore, although CXCL-10 levels were significantly higher in rejection compared with CMV infection at the group level in our cohort, individual-level overlap may still occur in larger or more heterogeneous clinical populations, underscoring the need to interpret CXCL-10 values in conjunction with clinical context and virological testing.

A recent meta-analysis by Udomkarnjananun et al. [40] evaluated the impact of tacrolimus exposure on clinical outcomes in kidney transplant recipients using contemporary, real-world data and confirmed an association between tacrolimus exposure and rejection risk, supporting the use of standard-dose tacrolimus in combination with mycophenolic acid to reduce acute rejection rates. Standard-dose tacrolimus exposure was associated with significantly lower acute rejection rates vs. reduced-dose exposure (odds ratio, 0.4 [95% confidence interval: 0.17, 0.95]; *p* = 0.037). However, no significant differences were observed in graft loss or patient survival, underscoring the complexity of translating trough concentrations into meaningful clinical decision-making. In addition, it is well known that patients with high variability in tacrolimus trough concentrations are at an increased risk of rejection, particularly when their mean tacrolimus C_0_ is in the lower part of the therapeutic range [41]. In our cohort, more than 80% of patients exhibited tacrolimus C_0_ concentrations within the recommended therapeutic range, and those with values below the target range did not show a higher incidence of rejection. Consistently, tacrolimus trough concentrations, measured either in venous WB or DBS, did not significantly differ between patients with rejection, CMV infection, or those free of clinical events, indicating that tacrolimus C_0_ alone did not provide diagnostic discrimination for rejection in this setting. These findings reinforce the concept that, although tacrolimus exposure is essential to ensure immunosuppressive safety and efficacy, monitoring based solely on trough concentrations has inherent limitations and does not reliably reflect the immunological status of the graft. These highlights added clinical value of integrating the measurement of CXCL-10, as pharmacodynamic biomarker, and supports the relevance of AUC estimation, in specific clinical situations, to provide complementary information beyond conventional tacrolimus trough monitoring.

Regarding tacrolimus measurement, the good correlation observed between tacrolimus concentrations measured in DBS and WB (reference method) confirms that DBS sampling offers a reliable alternative to conventional venipuncture for TDM. Linear regression analysis showed strong agreement with minimal bias, supporting its applicability in clinical practice. Moreover, in the validation cohort, both the Passing–Bablok regression and Bland–Altman analysis further demonstrated excellent concordance between matrices. This concordance strongly supports the analytical interchangeability between capillary DBS and venous WB measurements. The combined evidence from Passing–Bablok and Bland–Altman analyses demonstrates that DBS provides tacrolimus capillary blood concentrations that are equivalent to those obtained from venous WB, without clinically relevant systematic or proportional bias. These findings reinforce the methodological robustness of our approach and confirm DBS as a valid, reliable, and analytically sound alternative for tacrolimus TDM in routine clinical practice. Furthermore, it is important to highlight that this interchangeability provides a strong methodological basis for extending tacrolimus monitoring beyond single trough concentrations toward AUC-based exposure assessment. In this context, DBS sampling offers clear practical advantages by enabling repeated, minimally invasive sampling, which may facilitate individualized AUC estimation in both routine care and selected clinical scenarios, thereby supporting more personalized and patient-friendly immunosuppression management.

The analytical workflow was designed to ensure standardized DBS sampling by applying a fixed volume of capillary blood and controlling factors that may affect blood spreading within the predefined area of the card, such as hematocrit, blood sample applied, spot homogeneity, drying conditions, and paper characteristics. Entire spots were subsequently processed for sample preparation, analyte extraction, and LC-MS/MS analysis. The validation results demonstrated good precision, a suitable LLOQ and a linear response across the clinically relevant concentration range, in accordance with EMA bioanalytical validation criteria. These results make this LC-MS/MS method suitable for the determination of tacrolimus in clinical practice.

The DBS approach offers multiple practical advantages: minimally invasive, relatively painless, and requiring only a small volume of capillary blood that can be obtained via finger prick. Moreover, DBS collection can be performed by patients at home, reducing the need for hospital visits and enabling remote therapeutic and immunological monitoring. In contrast to DPS, as used in previous CXCL-10 studies of infectious diseases such as tuberculosis, DBS does not require prior sample manipulation such as centrifugation or plasma separation, procedures that must be performed under controlled laboratory conditions, which therefore limit patient self-collection. Therefore, DBS offers the unique advantage of being fully compatible with patient self-sampling, unlike DPS. Moreover, DBS cards are stable at room temperature, can be shipped by regular mail, and can be stored for extended periods without significant analyte degradation, making them particularly suitable for decentralized or resource-limited settings. From a methodological point of view, the response time is minimal; therefore, the results could be available in less than 48 h. Although DBS sampling has clear advantages, some technical limitations must be considered. Factors such as hematocrit variability, spot size, and filter paper type can influence analyte recovery and quantification. However, in our methodology, the whole spot is used for tacrolimus measurement. In addition, our study population comprises clinically stable kidney transplant recipients in the maintenance phase, who typically present a mean hematocrit value of approximately 35%. These sources of variability have minimal impact on assay performance.

Nevertheless, this study has several limitations that should be considered. First, it was a single-centre pilot study with a modest sample size, which limits the statistical power and generalizability of the results. Second, BKV infection was not assessed, precluding evaluation of its influence on CXCL-10 levels and its differentiation from other inflammatory causes. Third, while the precise degradation kinetics of CXCL-10 in DBS during the initial 1–2 weeks remain partially characterized, the absence of a dedicated stability study in this work represents a limitation that should be considered. Finally, despite the use of validated analytical methods for tacrolimus and CXCL-10, the cross-sectional design did not allow assessment of longitudinal biomarker dynamics or predictive performance for the assessment of the risk of rejection or infection.

This pilot study provides proof of concept that DBS-based measurement of CXCL-10 and tacrolimus is feasible and clinically meaningful. Combined pharmacokinetic (tacrolimus) and pharmacodynamic (CXCL-10) assessments could support personalized immunosuppression adjustment and early identification of rejection (TCMR or ABMR) as well as CMV infection. Future studies including a larger number of patients are warranted to refine diagnostic cut-offs, evaluate predictive performance (ROC-AUC, sensitivity, specificity, PPV, and NPV), and determine the role of DBS-CXCL-10 monitoring in longitudinal post-transplant surveillance.

## 4. Materials and Methods

### 4.1. Patients

An observational, retrospective cohort study was conducted at the Renal Transplant Unit of the Hospital Clinic of Barcelona. Biomarker analysis and immunosuppressive monitoring were performed at the Pharmacology and Toxicology Laboratory. The participants were kidney transplant recipients from deceased or living donors who received tacrolimus as an immunosuppressive treatment to prevent rejection. The study protocol was conducted in accordance with the Declaration of Helsinki, and the protocol was approved by the Ethics Committee of the Hospital Clinic of Barcelona (HCB/2019/0098, approved on 4 January 2019). All participants were informed about the study objectives and provided written informed consent prior to inclusion.

To assess CXCL-10 measurement in DBS using ELISA, a total of 81 adult kidney transplant recipients at the Renal Transplant Unit of the Hospital Clinic, from February 2022 to July 2023, and 10 healthy volunteers (used as controls) were included. Demographic and laboratory data are summarized in Table 1.

To validate the DBS-LC-MS/MS-based method for tacrolimus and evaluate its clinical applicability and feasibility for routine and home monitoring in stable kidney transplant recipients, two study cohorts were established from January 2019 to July 2023.

#### 4.1.1. Method Development and Validation Group for Tacrolimus DBS

The first cohort included 160 kidney transplant recipients enrolled for the validation of tacrolimus quantification in DBS samples between January 2019 and April 2023.

#### 4.1.2. Clinical Study Group (Maintenance-Phase Patients) for Tacrolimus DBS

The second cohort comprised clinically stable kidney transplant recipients (>12 months post-transplant), from deceased or living donors, under maintenance immunosuppression with tacrolimus to prevent rejection. Two sampling modalities were evaluated in this group: nurse-collected samples, in which tacrolimus measured from DBS was collected by trained nursing staff (n = 20); and self-collected samples, in which tacrolimus measured from DBS was collected by the patients at home (n = 16).

### 4.2. CXCL-10 Detection in DBS

CXCL-10 concentrations in DBS samples were measured using an adapted commercial ELISA kit (R&D Systems, Minneapolis, MN, USA). Capillary blood (25 μL) was applied to the designated spots of Whatman FTA™ DMPK-C cards and was allowed to dry for at least 12 h (overnight, O/N) at room temperature. DBS samples were promptly transferred to cold storage after drying to minimize potential degradation risks and were processed within 48 h. Two punched discs from each DBS sample were placed into individual wells of a 96-well ELISA plate, and 125 μL of assay diluent (RD1-56) was added to both standards and samples. Care was taken to ensure proper disc stacking and removal of air bubbles. Samples and standards were incubated for 2 h at room temperature, followed by incubation with conjugate buffer. After washing and disc removal, substrate solution was added, and the reaction was stopped after 30 min of incubation in the dark. Absorbance was measured at 450 nm using a microplate reader. Representative ELISA calibration curves (CXCL-10 concentrations: 500, 250, 125, 62.5, 31.2, 15.6, 7.8, and 0 pg/mL) used for CXCL-10 quantification in DBS samples are provided in the Supplementary Materials (Figure S1). When CXCL-10 concentrations exceeded the upper LQ, samples were appropriately diluted with assay diluent and reanalyzed to ensure that all measurements fell within the validated calibration range.

### 4.3. Tacrolimus Quantification in DBS Based Extraction and LC-MS/MS Analysis

The TDM of tacrolimus is usually based on the trough concentration (C_0_) measured in venous WB just before the drug is taken.

#### 4.3.1. Chemicals and Reagents

Lyophilized calibrators (six levels of concentrations: 1 ng/mL; 2.5 ng/mL; 5 ng/mL; 10 ng/mL; 20 ng/mL and 45 ng/mL), quality controls [3 ng/mL (Quality Control Low, QCL), 7.5 ng/mL (Quality Control medium, QCM), and 15 ng/mL (Quality Control High, QCH)] and IS were purchased in Waters (Recipe brand, 9933 and 8830 references respectively).

The IS contains ^13^Cd2-Tacrolimus, d12-CyclosporinA, ^13^Cd3-Sirolimus and ^13^C2d4-Everolimus with reference MS1412. Methanol and formic acid for LC-MS purity were bought in Honeywell (Barcelona, Spain) and acetonitrile LC-MS purity was bought in Merck (Barcelona, Spain); ammonium acetate was purchased from Sigma-Aldrich (Madrid, Spain). For DBS collection, we used an automatic lancet single-use Accu-check, Safe-T-Pro plus (Roche, Sant Cugat del Vallés, Barcelona, Spain), 25 µL capillary end-to-end microsafe pipette (Microsafe, Safetec) (Tens Medical Services, Central House, Gate Lane, Boldmere, Sutton Coldfield, West Midlands), and Whatman FTA^TM^ DMPK-C Cards (Sigma-Aldrich).

#### 4.3.2. DBS Sample Collection

Capillary blood samples were collected immediately before the morning tacrolimus dose under steady-state conditions (unchanged dose for at least three days). After finger disinfection with alcohol at 96 °C and drying, capillary blood flow was increased by applying a massage to the finger. After that, the finger was pricked with a single-use automatic lancet, and the first drop was discarded. Then, the second drop was collected by a 25 µL capillary end-to-end microsafe pipette until it was completely filled and the capillary was emptied in the marked circle of an Eastern Business Forms, Inc CE card (ref.10550097). The 25 µL placed on the Whatman FTA^TM^ DMPK-C card was allowed to dry for at least 12 h (O/N).

#### 4.3.3. Sample Extraction

After complete drying, two DBSs (25 μL each) were placed into a 2 mL tube, and 250 μL of the extraction solvent [MeOH/Ms-water (85/15, (*v*/*v*)) with deuterated IS)] was added for analyte extraction. Samples were vortexed for 1 min and centrifuged for a 20 s pulse. Then, they were sonicated for 20 min at 40 °C, vortexed for 1 min and centrifuged for a (pulse of about 15 s). The extract was transferred to a borosilicate tube, placed in a freezer for 10 min at −20 °C, centrifuged for 12 min at 4.000 g, and the supernatant transferred to UPLC vials. A volume of 20 μL was injected into the LC-MS/MS system.

#### 4.3.4. Equipment and Analytical Method

A method for the determining the immunosuppressant tacrolimus in dry blood on cardboard (DBS) was developed and validated in the Pharmacology Laboratory of the Hospital Clinic of Barcelona. The drug is extracted from the WB spot and subsequently analyzed by ultra-high efficiency liquid chromatography (UPLC) using an Acquity^TM^ Ultra Performance LC Waters coupled with a Triple Quadrupole mass spectrometer detector (Waters Cromatografía, S.A., Cerdanyola del Vallès, Barcelona, Spain). MassLynx 4.1 software version 1.40.2532 was used to acquire and analyze data (Waters).

Chromatographic separation was achieved using an ACQUITY UPLC^®^BEH C18 1.7 µm 2.1 × 30 mm, Waters analytical column (Waters) at 55 °C. Mobile Phase: 2 mM ammonium acetate + 0.1% with formic acid in water (phase a)/ammonium acetate 2 mm + 0.1% with methanol and formic acid (phase b) initial conditions: 0′–0.2′ (50% a/50% b), 0.2′–0.6′ (100% b), 0.6′–1.2′ (50%/50%). Flow: 0.4 (mL/min). Analytical column: ACQUITY UPLC^®^BEH C18 1.7 µm 2.1 × 30 mm, Waters (ref.186002349) (Cerdanyola del Vallès, Spain). Injection volume: 20 µL. Elution time: 2 min. Column temperature: 55 °C. Sample temperature: 10 °C. When tacrolimus concentrations exceeded the upper LQ, samples were appropriately diluted and reanalyzed to ensure that all measurements fell within the validated calibration range.

The MS conditions were optimized as follows: ESI positive polarity; nitrogen as nebulizer gas; capillary voltage: 1.2 kV; cone voltage: 30 V; source temperature: 130 °C; desolvation temperature: 350 °C; desolvation gas flow: 900 L/h; collision gas flow: 0.3 mL/min. Two ion transitions were monitored for each analyte. The mass transitions followed during the analysis are as follows: Tacrolimus: 821.6 to 768.4; ^13^Cd2-Tacrolimus: 824.5 to 771.5. Representative chromatograms for tacrolimus and the IS obtained from DBS samples are provided in the Supplementary Materials (Figure S2).

### 4.4. Statistical Analyses

Statistical analysis was performed using SPSS software, version 23.0 (SPSS Inc., Chicago, IL, USA). Data distributions were assessed for all continuous variables. Variables with non-normal distribution are presented as median and interquartile range (IQR), whereas normally distributed variables are expressed as mean ± standard deviation (SD). Statistical differences between groups were assessed with the Mann–Whitney test for pairwise comparisons (e.g., tacrolimus WB vs. DBS) whereas Kruskal–Wallis tests were applied for comparisons among more than two independent groups (e.g., CXCL-10 levels across clinical subgroups). Correlations between tacrolimus concentration in venous blood vs. DBS were assessed using linear regression followed by Passing–Bablok regression and Bland–Altman analysis. These methods evaluate method comparability without assuming normal distribution of errors and are robust to outliers. The regression estimates the intercept and slope with 95% confidence intervals to detect the presence of systematic or proportional bias between matrices. A *p* value ≤ 0.05 was considered statistically significant.

The diagnostic performance of DBS-CXCL-10 concentration for identifying rejection (TCMR + ABMR n = 22) was evaluated by calculating the area under the ROC curve (AUROC) and its 95% confidence interval (95% CI). The optimal cutoff point was determined using Youden’s index, defined as Max (sensitivity + specificity − 1). Based on this cutoff, sensitivity, specificity, PPV, and NPV were subsequently estimated.

## 5. Conclusions

Quantification in capillary blood using the DBS approach is feasible in kidney transplant recipients, supporting its potential use as a minimally invasive, decentralized tool for personalized immunosuppressive monitoring and for improving the clinical outcome. In parallel, tacrolimus concentrations measured in DBS showed excellent agreement and analytical interchangeability with conventional venous WB measurements, confirming DBS as a reliable alternative for tacrolimus TDM. The integration of CXCL-10 and tacrolimus monitoring from a single DBS card represents a promising step toward patient-centred, precision medicine in kidney transplantation.

## Figures and Tables

**Table 1:** Characteristics of study cohort for CXCL-10-DBS.

	Free CE	TCMR	ABMR	CMV
Recipients	53	12	10	6
Age at KTx (years)	51.6 ± 14.3	55.8 ± 19.7	55.2 ± 12.3	61.8 ± 7.7
Female n (%)	19 (35.8)	4 (33.3)	4 (40)	3 (50)
Number of KTx = 1 (%)	41 (77.4)	8 (66.7)	4 (40)	4 (66.7)
Etiology				
-Hereditary kidney disease	9	2	0	3
-Primary reflux nephropathy	6	1	3	0
-IgA	6	0	1	0
-Diabetic nephropathy	13	0	1	1
-Other	12	6	2	1
-Unknown etiology	7	3	3	2
Donor age (years)	55.6 ± 15.5	62.3 ± 15.4	51.89 ± 13.8	65.8 ± 7.2
Donor sex (Female)	23	4	3	4
Living donor	10	1	4	0
DCD—Maastricht II	0	2	0	0
DCD—Maastricht III	18	5	4	2
DBD	25	4	2	4
Time between sample collection and KTx (Median (CI), days)	372.5 (87–978)	102 (17–370)	253 (27–4149)	240 (180–418)

KTx, kidney transplantation; Hereditary kidney disease includes ADPKD, Alport and nephrotic syndrome; DCD, Donation after Circulatory Death; DBD, Donation after Brain Death; CE, clinical event; TCMR, T-cell mediated rejection; ABMR, antibody-mediated rejection and CMV, cytomegalovirus. Age data is presented as mean ± standard deviation (SD).

## Data Availability

The original contributions presented in this study are included in the article and Supplementary Materials. Further inquiries can be directed to the corresponding authors.

## Supplementary Materials

The following supporting information can be downloaded at: https://www.mdpi.com/article/10.3390/ph19020292/s1, Figure S1: Representative ELISA calibration curve for CXCL-10 quantification in DBS; Figure S2: Representative chromatograms for tacrolimus and the internal standard obtained from DBS samples; Table S1: Intra-day and inter-day precision and accuracy of the DBS LC–MS/MS method for tacrolimus quantification.
