# Supplementary material for: Dried Blood Spot for CXCL-10 and Tacrolimus: Integrated Non-Invasive Monitoring to Guide Personalized Treatment in Adult Kidney Transplant Recipients
Source: Pharmaceuticals (Basel). 2026 Feb 10;19(2):292. doi: 10.3390/ph19020292 (PMC12944364; doi:10.3390/ph19020292)
Supplement: Supplementary file 1 [file pharmaceuticals-19-00292-s001.zip › pharmaceuticals-4086999-supplementary.pdf]

Table S1.

Intra-day and inter-day precision and accuracy of the DBS LC–MS/MS method for tacrolimus quantification .

ACCURACY	INTRA-DAY ASSAY %CV	INTER-DAY ASSAY %CV
LLQC: 1ng/mL	5.50	7.10
QCL: 3 ng/mL	-0.80	-0.80
QCM: 7.5 ng/mL	-1.17	-1.10
QCH: 15 ng/mL	-3.77	-3.70
**PRECISION**	**INTRA-DAY ASSAY %CV**	**INTER-DAY ASSAY %CV**
LLQC: 1ng/mL	5.30	7.70
QCL: 3 ng/mL	4.27	4.20
QCM: 7.5 ng/mL	3.43	3.90
QCH: 15 ng/mL	3.40	3.50

Figure S1.

Representative ELISA calibration curve for CXCL-10 quantification in DBS

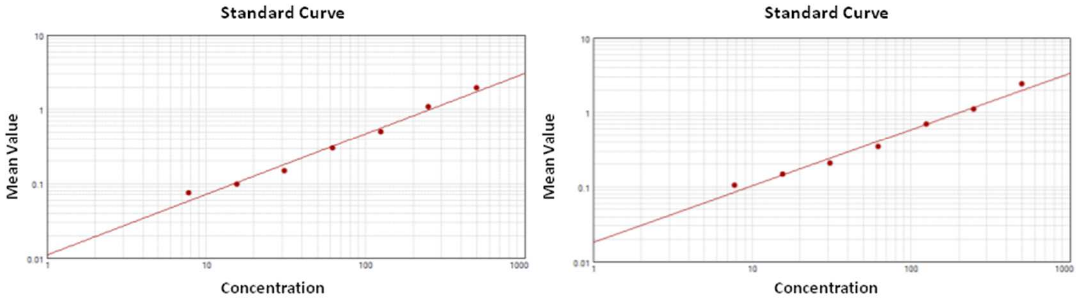

Standard	Concentration (pg/mL)	BackCalConc	Value
1	500	569.966	1.925
2	250	282.329	1.087
3	125	108.698	0.500
4	62.5	57.071	0.296
5	31.2	24.351	0.148
6	15.6	14.594	0.098
7	7.8	10.451	0.074
8	0	9.678	0.070

Standard	Concentration (pg/mL)	BackCalConc	Value
1	500	539.556	1.822
2	250	230.884	1.104
3	125	126.163	0.699
4	62.5	49.286	0.344
5	31.2	25.357	0.208
6	15.6	15.774	0.145
7	7.8	10.095	0.104
8	0	8.418	0.090

Figure 1S

Figure S2.

Representative chromatograms for tacrolimus and the internal standard obtained from DBS samples

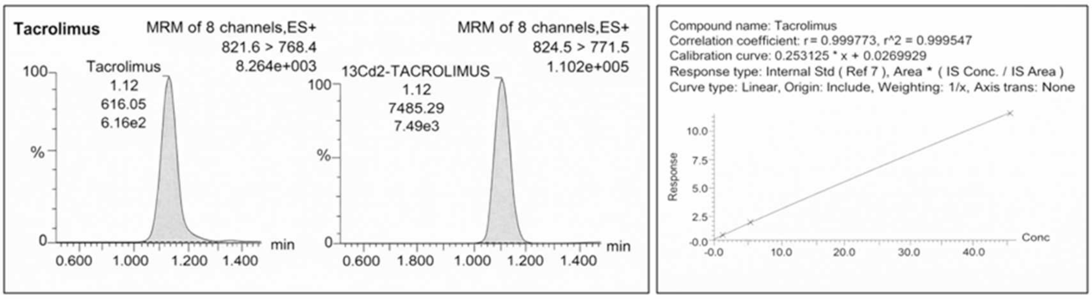

Name	Trace	RT	Area	IS Area	Response	Detection	ng/mL	%Dev
Tacrolimus	821.6>768.4	1.12	616.055	7485.286	1.029	MM	4.0	
13Cd2-Tacrolimus	824.5>771.5	1.12	7485.286		7485.286	MM	12.8	2.6

Figure 2S
